# Evaluating the psychometric properties of the Persian version of the Healthy Lifestyle Instrument for Breast Cancer Survivors (HLI-BCS)

**DOI:** 10.1186/s12905-023-02208-3

**Published:** 2023-02-09

**Authors:** Elahe Ramezanzade Tabriz, Seyed Amir Aledavood, Monir Ramezani, Fateme Kavoosi

**Affiliations:** 1 Department of Medical Surgical Nursing, School of Nursing and Midwifery, Mashhad University of Medical Sciences, Mashhad, Iran; 2 Cancer Research Center, Mashhad University of Medical Sciences, Mashhad, Iran; 3 Nursing and Midwifery Care Research Center, Mashhad University of Medical Sciences, School of Nursing and Midwifery, P.O. Box 9137913199, Ebne Sina St, Mashhad, Iran

**Keywords:** Lifestyle, Breast cancer, Survivors, Psychometrics

## Abstract

**Background:**

Precise examination of breast cancer survivors’ lifestyles can lead to improved planning and implementation of care and counseling interventions to increase their survival rate and improve their quality of life. Therefore, a valid and reliable instrument needs to be developed. Therefore, the present study aimed to determine the psychometric properties of the Persian version of the Healthy Lifestyle Instrument for Breast Cancer Survivors (HLI-BCS).

**Methods:**

This methodological study was conducted on 420 Iranian breast cancer survivors between May and November 2022. Participants were selected using convenience sampling. The face, content, construct validity, convergent, and reliability of the Persian version of the HLI-BCS were assessed.

**Results:**

After assessing face, content, and construct validity, the Persian version of the HLI-BCS with five factors and 20 items was provided. The total Cronbach’s alpha and intra-class correlation coefficient (ICC) were calculated as 0.86 and 0.79, respectively, which were at acceptable levels. A healthy lifestyle in breast cancer survivors was observed to have strong and significant relationships with quality of life in general (*p* < 0.001, r = 0.832), physical health (r = 0.786), and mental health (r = 0.809).

**Conclusion:**

The Persian version of the HLI-BCS has favorable properties, is consistent with the conditions of breast cancer survivors, and is valid and reliable. This version of the scale can provide adequate and precise information on the lifestyles of these patients.

## Background

Breast cancer is the most common invasive cancer among women and involves one in every eight women. It is considered the leading cause of cancer-related deaths among women worldwide, including in Iran [1, 2].

The importance of breast cancer as a disease with high incidence and mortality rates is thought-provoking, particularly in developing countries, because 58% of deaths occur in less-developed countries [3]. In addition, studies have shown that increasing life expectancy and the aging index increase the incidence of breast cancer in the years to come. However, timely diagnosis, treatment adherence, and improved quality of life can enhance the survival rate of infected individuals and prevent human and financial capital losses [4].

Therefore, given the high prevalence and survival rate of breast cancer, the importance of status examination, treatment follow-up, and lifestyle of individuals with this cancer after completing the treatment is emphasized. Cancer survivors further adhere to self-care recommendations to improve long-term treatment outcomes compared to the time of diagnosis [5, 6]. Moreover, for many cancer survivors with high survival rates, weight management, a healthy diet, and an active lifestyle are priorities for preventing the recurrence of primary cancers and other chronic diseases [7]. Improving lifestyle is highly important in the period after a cancer diagnosis because it is the time when survivors perceive the importance of changing their lifestyle and can change their behaviors, complete their primary treatment, including surgery and chemotherapy, and seek a healthy lifestyle to prevent repeated recurrence and promote survival [8, 9].

One way to check an individual’s healthy lifestyle is to measure elements that are considered as the lifestyle’s objective manifestations [6]. Instruments can collect data with the highest precision and accuracy, and help healthcare providers, including doctors, nurses, and paramedics, properly identify patients’ needs, prioritize their care, and provide them with the best services in the most appropriate situation [10]. Hence, a precise instrument must be specifically designed to evaluate the lifestyle of patients with breast cancer, determine their status, and improve their lifestyle by providing educational and care services.

Currently, there is a dedicated Healthy Lifestyle Instrument for Breast Cancer Survivors to evaluate their post-treatment status. The Healthy Lifestyle Instrument for Breast Cancer Survivors (HLI-BCS) was designed by Wang et al. [11] and used in a Taiwanese population. It has favorable psychometric properties.

Therefore, given the high prevalence of breast cancer in Iran, a standard and indigenous lifestyle assessment instrument for planning and providing care interventions for breast cancer survivors has become increasingly evident. Also, in Iranian society, there is no indigenous Persian version of the instrument for patients with breast cancer, consistent with Iranian Islamic culture. Therefore, this study was conducted to determine the psychometric properties of the Persian version of the HLI-BCS in Iranian society.

## Methods

The current methodological study was carried out on female breast cancer survivors referred to hospitals affiliated with Mashhad University of Medical Sciences from May to November 2022.

### Study sample

The sample used in this study was selected through convenience sampling. Women with breast cancer had the following inclusion criteria: a history of at least two years of breast cancer, having no recurrent or metastatic disease, receiving treatment in one of the stages of surgery, chemotherapy, or radiotherapy, consenting to participate in the study, and having the ability to answer the questionnaire items. The exclusion criteria were no mental disorder, not using psychiatric medications, and reluctance to participate in the study. According to Kellar and Kelvin (2012), the minimum sample size to perform factor analysis is five to ten times higher than the number of items in the intended instrument [12, 13]. Thus, the Persian version of the HLI-BCS contains 20 items: 420 patients with breast cancer participated in two stages, including 220 samples for confirmatory factor analysis (CFA) and 200 patients meeting the inclusion criteria, to assess the convergent validity between the Persian versions of the HLI-BCS and the 12-item Short Form Health Survey (SF-12).

### Study instruments

#### The Demographic Information Questionnaire

Data were collected using the demographic information form, Persian version of the HLI-BCS, and SF-12. The demographic information form included age, marital status, education level, employment status, economic status, health insurance, type of treatment, cancer stage, and duration of breast cancer.

#### The Healthy Lifestyle Instrument for Breast Cancer Survivors (HLI-BCS)

HLI-BCS was developed by Wang et al in 2015 [11]. This instrument contains 20 items in five areas: dietary habits (items 1–5), environment and physiology (items 6–8), health responsibility and stress management (items 9–13), social and interpersonal relations (items 14–16,) and spiritual growth (items 17–20). Through the information obtained by filling out the instrument, the lifestyles of breast cancer survivors can be evaluated in different dimensions, and plans can be made to improve their lifestyles and quality of life. Of the instrument items, 13 had negative expressions and seven had positive expressions. The scoring of the items was based on a 5-point Likert scale from 1 to 5 [1 (never), 2 (relatively), 3 (sometimes), 4 (often), and 5 (always)]. The total score on the numerical instrument ranged from 20 to 100. In the psychometrics of the original version of the instrument, face validity and content validity were confirmed by 10 patients with at least five years of disease and five oncologists with ten years of experience working with patients with breast cancer. CFA has confirmed the instrument’s construct validity on 230 breast cancer survivors in the Taiwanese population, and the mean Cronbach’s alpha for the instrument’s dimensions was 0.8 [10].

#### The Iranian version of 12-item Short Form Health Survey (SF-12)

The SF-12 is the short form of the 36-item Short Form Health Survey (SF-36), designed by Ware et al. in 1996. For the reliability of the questionnaire, the calculated Cronbach’s alphas for the physical and mental dimensions were 0.89 and 0.76, respectively, showing the favorable reliability of the questionnaire items [14]. Montazeri et al. [15] published the Iranian version of SF-12 containing 12 items in two physical and mental health dimensions. The present questionnaire investigated the quality of life regarding the general perception of one’s health, physical performance, physical health, emotional problems, physical pain, social performance, vitality, and perceived mental health. The scores obtained by the participants showed good status (37–48), moderate status (25–36), and poor status (12–24). The reliability of the Iranian version in physical and mental dimensions was reported as 0.73 and 0.72, respectively [15].

### Translation

The translation was performed using a forward–backward translation protocol from English to Persian using the World Health Organization (WHO, 2016) method [16]. Two English-to-Persian translators were asked to independently translate the questionnaire. A Persian-to-English translator was then asked to re-translate the Persian questionnaire into English. It was then sent to five experts in Persian language and literature, English language, instrument design, and nursing for final approval to ensure the precise transfer of concepts and proper translation of the translated instrument. After receiving these comments, an initial Persian version was prepared.

### Analysis methods

#### Face and Content validity

A cognitive interview and pretest were performed with the target group to ensure translation validity. Accordingly, ten patients with breast cancer who met the criteria to fill out the questionnaire were interviewed individually regarding the clarity of every single item and the precise measurement of the real variable using the Persian instrument. The target group was asked to express their opinions and suggested words to better understand each item or to replace words [17].

A group of 15 experts calculated the content validity ratio (CVR) and content validity index (CVI) to assess the relevance, clarity, and simplicity of the items. The group of experts consisted of faculty members from the nursing department, oncologists, oncology nurses, oncology surgeons, clinical nutritionists, psychiatrists, and social workers. When the number of experts was 15, the minimum acceptable CVR was 0.49, based on Lawshe’s table [18]. The minimum acceptable CVI value for each item is 0.7 [19].

#### Construct validity

CFA was performed using the maximum likelihood estimation method and the most common goodness-of-fit indices for 220 participants to evaluate the structural factors of the Persian version of the HLI-BCS. Model fit indices were evaluated according to (2 < χ^2^/*df* < 5), the root mean square error of approximation (RMSEA < 0.08), the comparative fit index (CFI > 0.9), the goodness of fit index (GFI > 0.9), parsimonious normed fit index (PNFI > 0.5), and the Tucker–Lewis index/non-normed fit index (TLI/NNFI > 0.9). Items with factor loadings less than 0.4 were removed from the model [20, 21].

#### Convergent validity

The correlation between the results of the Persian version of the HLI-BCS and the SF-12 was compared to evaluate the convergent validity of the questionnaire. The Pearson’s correlation coefficient was used for the convergent validity test. Pearson correlation assumes that the variables are parametric; both variables should be quantitative and interval/ratio, and the data should have a normal distribution. In this study, the normality of the data was determined based on a kurtosis between ± 7 and skewness between ± 3. Therefore, a Pearson’s correlation coefficient of ≥ 0.40 was considered acceptable (17).

#### Reliability

Internal consistency was examined using Cronbach’s alpha to check instrument reliability. Cronbach’s alpha ranged from 0 to 1. Higher values indicate higher reliability, and a minimum level of 0.7 is recommended for alpha. In addition, the test–retest method and intraclass correlation coefficient (ICC) were used to determine reliability, and 30 patients who met the criteria for completing the instrument responded to the questions of the Persian version within two weeks. The ICC rate is between zero and one, the reliability coefficient of 0.6 is acceptable, and reliability coefficients of 0.8 and higher denote the instrument’s great stability [17, 22].

### Multivariate normality and outliers

Univariate and multivariate distributions were assessed for skewness and kurtosis, and Mardia’s coefficient for outliers. One of the signs of deviation from the normal distribution was a Mardia’s coefficient > 8. Multivariate outliers were evaluated by assessing the Mahalanobis distance. Items with a Mahalanobis distance of *p* < 0.001 were considered as multivariate outliers. All statistical analyses were performed using IBM SPSS Statistics version 24 and IBM SPSS Amos version 25. A significance level of *p* < 0.05 was considered for all statistical tests [23].

## Results

### Socio-demographic and clinical status

Four hundred and twenty women with breast cancer participated in this study (mean age = 47.63 ± 11.08 years). Most participants (83.1%) were married, 71.6% had an under-diploma education, and the mean number of years of disease was 3.37 ± 1.27. Other demographic, social, and cancer-related information about the patients is provided in Table 1.

**Table 1:** Socio-demographic and cancer information of participants (n = 420)

Characteristics		Mean (SD) or n (%)
Age range (Year)		47.63 (11.08)
Marital status n (%)	Single	29 (6.9)
	Married	349 (83.1)
	Divorced	23 (5.5)
	Widow	19 (4.5)
Education status n (%)	Under diploma	301 (71.6)
	Diploma	88 (21)
	Academic	31 (7.4)
Occupational status n (%)	Unemployed	304 (72.4)
	Employed	72 (17.2)
	Retired	44 (10.5)
Economic status n (%)	Poor	307 (73.1)
	Moderate	101 (24.9)
	Good	12 (2.9)
Insurance n (%)	Yes	385 (91.7)
	No	35 (8.3)
Time since diagnosis (Year)		3.37 (1.27)
Treatment type n (%)	Surgery	30 (7.1)
	Surgery and chemotherapy	135 (32.1)
	Surgery and radiotherapy	19 (4.5)
	Combination of treatments	236 (56.3)
Stage of disease n (%)	I	101 (24)
	II	191 (45.5)
	III	122 (29)
	IV	6 (1.5)
History of comorbidity n (%)	Yes	294 (70)
	No	126 (30)

*SD* standard deviation

### Face and content validity

After completing the translation process, the face validity of the items in the Persian version of the questionnaire was approved by 10 patients with cancer. Understanding the difficulty of the items, understanding the expressions, and the items’ apparent fit with the central objective of the questionnaire was acceptable for the patients. In addition, none of the items underwent semantic changes owing to semantic differences, and all items entered the content validity process.

Regarding CVR, obtaining scores lower than acceptable values in Lawshe’s table for 15 experts (less than 0.49) omitted the items, but all items with acceptable CVRs were between 0.8 and 1. The CVIs of all items were acceptable (0.7<). Finally, S-CVI/Ave was calculated to be 0.92.

### Construct validity

Skewness, kurtosis, and Mardia’s coefficient of the data were assessed to determine the CFA type. The skewness values of all the variables were between −3 and + 3, and their kurtosis values were between − 10 and + 10. Therefore, an assumption of normality was established.

**Fig. 1:**
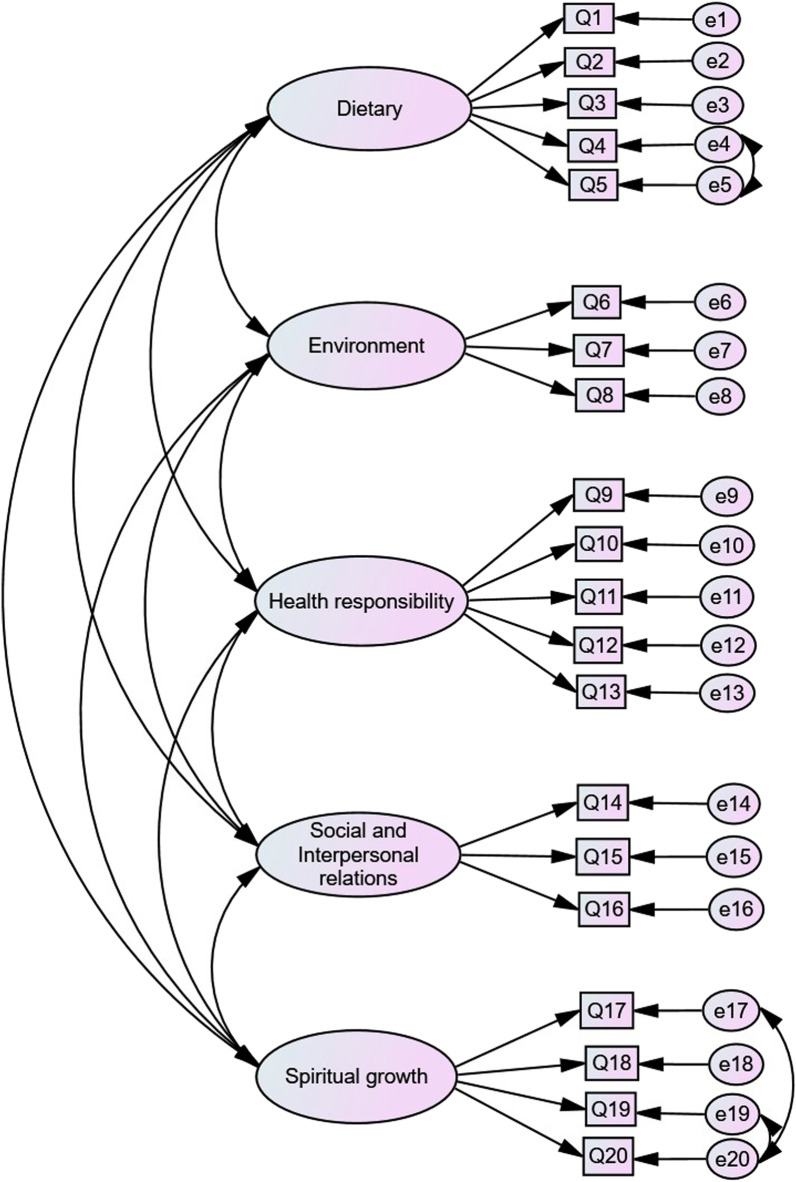
Path diagram of a confirmatory factor analysis of Persian version of HLI-BCS

CFA was performed on a sample of 220 patients. The χ^2^/*df* and fit indices indicated that the five-factor model of the Persian version of the HLI-BCS had a good fit for patients with breast cancer (Table 2). After providing the correction index, the model shown in Fig. 1 was confirmed.

**Table 2:** The fit model indices of the CFA for the Persian version of HLI-BCS (n = 220)

Fit indices	Acceptable scores	Scores
χ^2^/*df*	2 < χ^2^/*df* < 5	3.819
GFI (Goodness of fit index)	0.90 < GFI	0.921
CFI (Comparative fit index)	0.90 < CFI	0.913
TLI/NNFI (Tucker–Lewis index/Non normed fit index)	0.09 < TLI/NNFI	0.927
PNFI (Parsimonious normed fit index)	PNFI > 0.5	0.349
RMSEA (Root mean square error of approximation)	0.05 < RMSEA < 0.08	0.068

### Convergent validity

The mean and standard deviation of a healthy lifestyle in breast cancer survivors and their quality of life were 79.81 ± 22.07 and 33.04 ± 8.16, respectively. Pearson’s correlation coefficient was used to measure the correlation between the Persian version of the HLI-BCS and the SF-12. A healthy lifestyle in breast cancer survivors was observed to have strong and significant relationships with quality of life in general (*p* < 0.001, r = 0.832), physical health (r = 0.786), and mental health (r = 0.809) (Table 3).

**Table 3:** Correlations between the Persian version of HLI-BCS and SF-12 (n = 200)

Domains	Physical health	Mental health	SF-12 total score
Dietary habits	0.762	0.578	0.642
Environment and physiology	0.654	0.643	0.671
Health responsibility and Stress management	0.579	0.541	0.659
Social and interpersonal relations	0.674	0.932	0.813
Spiritual growth	0.681	0.853	0.902
HLI-BCS	0.786	0.809	0.832

All significant at *p* < 0.001

### Reliability

Cronbach’s alpha was calculated as the internal consistency criterion for the 20-item Persian version. The total questionnaire had high internal consistency with an alpha coefficient of 0.84, and the alpha coefficients for the subscales ranged from 0.66–0.86. The total relative stability using the ICC was also found to be 0.79.

## Discussion

This study aimed to evaluate the psychometric properties of the Persian version of the HLI-BCS among breast cancer survivors in Iran. The results of the current study support the five-factor structure of the HLI-BCS with 20 items in the dimensions of dietary habits (5 items), environment and physiology (3 items), health responsibility and stress management (5 items), social and interpersonal relations (3 items), and spiritual growth (4 items). The results indicated that the HLI-BCS had satisfactory validity and reliability with appropriate model fit indices, high levels of internal consistency reliability, and convergent validity to measure healthy lifestyles in breast cancer survivors in Iran.

To evaluate the validity of the Persian version of the HLI-BCS, face validity was used in breast cancer survivors, and content validity was used in a group of experts experienced in caring for cancer patients. The S-CVI/Ave index of 0.92 for the Persian version shows that the questionnaire’s content has been confirmed, considering the sociocultural status of breast cancer survivors in the Iranian community. Acceptable CVR and CVI for the Persian version indicate that the questionnaire’s content has appropriate quality, given the clinical characteristics of these patients in the Iranian community. In the original version of the questionnaire, content validity was assessed by five experts, but there needs to be a report on calculating CVR and CVI. In addition, HLI-BCS is yet to be assessed psychometrically in other communities.

The current research confirmed the structural factors of HLI-BCS in the Iranian community using CFA. The factors and 20 items were confirmed in the original version using CFA and favorable fit indices. The five factors of the Persian version of the HLI-BCS are dietary habits, environment and physiology, health responsibility, stress management, social and interpersonal relations, and spiritual growth. Dietary habits refer to the survivors’ dietary habits and preferences and the nutritional value of the selected foods. Environmental and physiological factors are related to perceived physiological problems and the perception of probable contamination sources in the current environment. Health responsibility and stress management factors indicate one’s perceived responsibility and the emotional and stressful reactions survivors face in their daily life or work. Social and interpersonal relationship factors reflect survivors’ general perceptions and feelings regarding social and interpersonal relations. The spiritual growth factor refers to survivors’ willingness to provide feedback and accept others in their lives [11, 24].

The convergent validity of the Persian version of the HLI-BCS was confirmed by its correlation with the SF-12 quality of life. A significant correlation was observed between a healthy lifestyle and quality of life scores in breast cancer survivors. In similar studies, having a healthy lifestyle, including adequate exercise, proper consumption of fruits and vegetables, weight loss, and satisfactory sleep, effectively improved the quality of life concerning the health of breast cancer survivors [25–28]. Improving quality of life can also be associated with favorable prognosis and lower mortality in breast cancer survivors [28].

In the current study, the dimensions of social and interpersonal relations and spiritual growth had the highest correlations with mental health. In addition, the total quality of life score was highly correlated with spiritual growth in the Iranian population with breast cancer. Some studies have indicated that increasing spiritual/religious activities and, in particular, increasing the frequency of attending spiritual/religious activities, the social support present in religious groups, and relaxation by attending homogeneous groups regarding the disease are related to improving health status [29–31]. In addition, promoting the power of resilience, along with different approaches to spiritual therapy, can lead to improved mental health and quality of life, improved psychological well-being and lifestyle, reduced depression, reduced negative emotions, and increased positive and creative thoughts in breast cancer survivors [31–33].

The internal consistency and stability of the scales were measured to determine their reliability. Internal consistency was evaluated by calculating Cronbach’s alpha coefficient. The alpha coefficient of the total scale was 0.84, which was acceptable. For all factors, the Cronbach’s alpha coefficient was higher than 0.7, indicating the consistency of the items in a factor and measuring similar properties. ICC was then calculated for the total scale to determine its stability. The ICC value was 0.79, indicating stability of the scale over time. In the HLI-BCS for internal consistency, a Cronbach’s alpha of 0.82 was obtained for the total instrument, and a test–retest reliability of 0.86, were obtained for the total instrument [11]. Therefore, considering the assessment of the original and Persian versions, reliability was calculated with an acceptable coefficient. In addition, the repeatability of the results over time and the consistency of the items’ dimensions were confirmed.

### Limitations

The first limitation of this study may be recall bias, because the data were collected using self-report questionnaires. Another limitation is that the validity and reliability of the Persian version is limited to a geographical region. It is better to conduct more methods of construct validity with more diverse samples to ensure strong psychometric properties of the instrument.

## Conclusions

Nurses are responsible for institutionalizing health in society worldwide, including Iran. For this purpose, they must create a culture in which individuals are unconsciously inclined toward healthy lifestyles. This effort requires a complex process based on a proper understanding of lifestyle and the relationships among various constituent elements of a healthy lifestyle. In other words, the prerequisite for achieving this goal is the ability of nurses to accurately recognize and assess how individuals behave and to compile an appropriate program based on a valid and reliable measurement. This study helped to develop a valid and reliable instrument with favorable characteristics compatible with the conditions of breast cancer survivors that can be used to provide adequate and precise information on the lifestyle of these individuals.

## Data Availability

The datasets used and/or analyzed during the current study are available from the corresponding author upon reasonable request.

## Abbreviations

HLI-BCS: Healthy Lifestyle Instrument for Breast Cancer Survivors
ICC: Intra-class correlation coefficient
CFA: Confirmatory factor analysis
SF-12: 12-Item Short Form Health Survey
WHO: World Health Organization
CVR: Content validity ratio
CVI: Content validity index
RMSEA: Root mean square error of approximation
CFI: Comparative fit index
GFI: Goodness of fit index
PNFI: Parsimonious normed fit index
TLI/NNFI: Tucker–Lewis Index/Non-normed fit index

## References

[1] Kashyap D, Pal D, Sharma R, Garg VK, Goel N, Koundal D (2022). Global increase in breast cancer incidence: risk factors and preventive measures. Biomed Res Int.

[2] Mottram R, Knerr WL, Gallacher D, Fraser H, Al-Khudairy L, Ayorinde A (2021). Factors associated with attendance at breast cancer screening: a systematic review and meta-analysis. BMJ Open.

[3] Francies FZ, Hull R, Khanyile R, Dlamini Z (2020). Breast cancer in low-middle income countries: abnormality in splicing and lack of targeted treatment options. Am J Cancer Res.

[4] Lei S, Zheng R, Zhang S, Wang S, Chen R, Sun K (2021). Global patterns of breast cancer incidence and mortality: a population-based cancer registry data analysis from 2000 to 2020. Cancer Commun (Lond).

[5] Blum D, Omlin A, Baracos VE, Solheim TS, Tan BH, Stone P (2011). Cancer cachexia: a systematic literature review of items and domains associated with involuntary weight loss in cancer. Crit Rev Oncol Hematol.

[6] Bull FC, Al-Ansari SS, Biddle S, Borodulin K, Buman MP, Cardon G (2020). World Health Organization 2020 guidelines on physical activity and sedentary behaviour. Br J Sports Med.

[7] Joseph R, Hart NH, Bradford N, Agbejule OA, Koczwara B, Chan A (2022). Diet and exercise advice and referrals for cancer survivors: an integrative review of medical and nursing perspectives. Support Care Cancer.

[8] Williams VA, Brown NI, Johnson R, Ainsworth MC, Farrell D, Barnes M (2021). A web-based lifestyle intervention for cancer survivors: feasibility and acceptability of SurvivorSHINE. J Cancer Educ.

[9] Tabriz ER, Ramezani M, Heydari A, Aledavood SA (2021). Health-promoting lifestyle in colorectal cancer survivors: a qualitative study on the experiences and perspectives of colorectal cancer survivors and healthcare providers. Asia Pac J Oncol Nurs.

[10] Mohammadbeigi A, Mohammadsalehi N, Aligol M (2015). Validity and reliability of the instruments and types of measurements in health applied researches. JRUMS.

[11] Wang HH, Chung UL, Tsay SL, Hsieh PC, Su HF, Lin KC (2015). Development and preliminary testing of an instrument to measure healthiness of lifestyle among breast cancer survivors. Int J Nurs Pract.

[12] Waltz CF, Strickland OL, Len ER (2017). Measurement in nursing and health research.

[13] Kellar SP, Kelvin EA, Munro BH (2012). Munro's statistical methods for health care research.

[14] Ware J, Kosinski M, Keller SD (1996). A 12 Item Short Form Health Survey: construction of scales and preliminary tests of reliability and validity. Med Care.

[15] Montazeri A, Vahdaninia M, Mousavi SJ, Omidvari S (2009). The Iranian version of 12 item Short Form Health Survey (SF 12): factor structure, internal consistency and construct validity. BMC Public Health.

[16] World Health Organization (WHO). Process of translation and adaptation of instruments. 2016. http://www.who.int/substance_abuse/research_tools/translation/en/. Accessed 30 Oct 2016.

[17] Polit DF, Yang F (2016). Measurement and the measurement of change: a primer for the health professions.

[18] Lawshe CH (1975). A quantitative approach to content validity 1. Pers Psychol.

[19] Zamanzadeh V, Ghahramanian A, Rassouli M, Abbaszadeh A, Alavi-Majd H, Nikanfar AR (2015). Design and implementation content validity study: development of an instrument for measuring patient-centered communication. J Caring Sci.

[20] Xia Y, Yang Y (2019). RMSEA, CFI, and TLI in structural equation modeling with ordered categorical data: the story they tell depends on the estimation methods. Behav Res Methods.

[21] Schreiber JB, Nora A, Stage FK, Barlow EA, King J (2006). Reporting structural equation modeling and confirmatory factor analysis results: a review. J Educ Res.

[22] Ramezanzade Tabriz E, Ramezani M, Manzari ZS, Jamali J, Heydari A (2022). Psychometric properties of the Persian version of the short-form survivor unmet needs survey (SF-SUNS) among patients with cancer. Asia Pac J Oncol Nurs.

[23] Çokluk Ö, Koçak D (2016). Using horn’s parallel analysis method in exploratory factor analysis for determining the number of factors. Educ Sci Theory Pract.

[24] Pender NJ, Murdaugh CL, Parsons MA (2014). Health promotion in nursing practice.

[25] Zheng C, Yu LX, Jia HY, Cui SD, Tian FG, Fan ZM (2021). Relationship between lifestyle habits and health-related quality of life of recently diagnosed breast cancer patients: a comparison between younger and older women in China. Front Public Health.

[26] Yusoff J, Ismail A, Abd Manaf MR, Ismail F, Abdullah N, Muhammad R (2022). Quality of life of women with breast cancer in a tertiary referral university hospital. Health Qual Life Outcomes.

[27] Di Meglio A, Soldato D, Presti D, Vaz-Luis I (2021). Lifestyle and quality of life in patients with early-stage breast cancer receiving adjuvant endocrine therapy. Curr Opin Oncol.

[28] Montagnese C, Porciello G, Vitale S, Palumbo E, Crispo A, Grimaldi M (2020). Quality of life in women diagnosed with breast cancer after a 12-month treatment of lifestyle modifications. Nutrients.

[29] Balboni TA, Vanderwerker LC, Block SD, Paulk ME, Lathan CS, Peteet JR (2007). Religiousness and spiritual support among advanced cancer patients and associations with end-of-life treatment preferences and quality of life. J Clin Oncol.

[30] Vallurupalli M, Lauderdale K, Balboni MJ, Phelps AC, Block SD, Ng AK (2012). The role of spirituality and religious coping in the quality of life of patients with advanced cancer receiving palliative radiation therapy. J Support Oncol.

[31] Zare A, Bahia NJ, Eidy F, Adib N, Sedighe F (2019). The relationship between spiritual well-being, mental health, and quality of life in cancer patients receiving chemotherapy. J Fam Med Prim Care.

[32] Jafari N, Farajzadegan Z, Zamani A, Bahrami F, Emami H, Loghmani A (2013). Spiritual well-being and quality of life in Iranian women with breast cancer undergoing radiation therapy. Support Care Cancer.

[33] Joshi AM, Mehta SA, Pande N, Mehta AO, Randhe KS (2021). Effect of Mindfulness-Based Art Therapy (MBAT) on psychological distress and spiritual wellbeing in breast cancer patients undergoing chemotherapy. Indian J Palliat Care.

